# New Section: Clinical Diagnosis and Prognosis

**DOI:** 10.3390/diagnostics14202258

**Published:** 2024-10-10

**Authors:** Andreas Kjaer

**Affiliations:** 1 Department of Clinical Physiology and Nuclear Medicine, Copenhagen University Hospital—Rigshospitalet, 2100 Copenhagen, Denmark; akjaer@sund.ku.dk; 2 Cluster for Molecular Imaging, Copenhagen University Hospital—Rigshospitalet & Department of Biomedical Sciences, University of Copenhagen, 2200 Copenhagen, Denmark

It is with pleasure that we introduce a new section of *Diagnostics*, named “Clinical Diagnosis and Prognosis”.

The target articles for this section are manuscripts with a focus on clinical diagnosis, stratification, therapy selection (companion diagnostics), and prognostication. Articles focused on developing and evaluating technologies in depth should continue to be submitted to more relevant sections, e.g., “Medical Imaging and Theranostics”, “Pathology and Molecular Diagnostics”, etc. This new section will focus on papers with a clinical focus on established methods.

We hope that with the addition of the section entitled “Clinical Diagnosis and Prognosis”, we can attract more manuscripts within the scope of *Diagnostics* and also make it more clear to authors where their manuscripts belong. However, the editors and editorial office of *Diagnostics* will naturally be assisting in allocating manuscripts to the section that best suits their subject matter.

I would also like to take this opportunity to thank all contributors for supporting our journal, which is not only currently thriving, but which we also expect to have an ever-increasing impact on the scientific community. Finally, I would like to thank the editors and editorial office for their excellent work in strengthening our journal’s handling of manuscripts, as well as their tireless work to increase our journal’s visibility and impact.

